# Nomogram Model for Dynamic and Individual Prediction of Cardiac Response and Survival for Light Chain Amyloidosis in 737 Patients With Cardiac Involvement

**DOI:** 10.3389/fonc.2021.758502

**Published:** 2021-12-09

**Authors:** Yang Li, Yanze Cao, Mingxin Zheng, Jiaqi Hu, Wei Yan, Xiaoyu Liu, Aijun Liao, Wei Yang, Jian Li, Huihan Wang

**Affiliations:** ^1^ Haematology Department of Shengjing Hospital, China Medical University, Shenyang, China; ^2^ Neusoft Research Institute, Northeastern University, Shenyang, China; ^3^ Department of Hematology, Peking Union Medical College Hospital, Chinese Academy of Medical Sciences & Peking Union Medical College, Beijing, China

**Keywords:** light-chain amyloidosis, cardiac involvement, nomogram model, cardiac response, overall survival

## Abstract

**Objective:**

Light chain amyloidosis (AL) with cardiac involvement is associated with poor prognosis. The existing prognostic assessment system does not consider treatment-related factors, and there is currently no effective system for predicting the response. The purpose of this study was to build an individualized, dynamic assessment model for cardiac response and overall survival (OS) for AL patients with cardiac involvement.

**Methods:**

The records of 737 AL patients with cardiac involvement were collected through cooperation with 18 hospitals in the Chinese Registration Network for Light-chain Amyloidosis (CRENLA). We used univariate and multivariate analyses to evaluate the prognostic factors for OS and cardiac response. Then, two nomogram models were developed to predict OS and cardiac response in AL patients with cardiac involvement.

**Results:**

A nomogram including four independent factors from the multivariate Cox proportional hazards analysis—Mayo staging, courses of treatment, hematologic response, and cardiac response—was constructed to calculate the possibility of achieving survival by adding all the points associated with four variables. The higher the score, the more likely death would occur. The other nomogram model included the courses of treatment, hematological response, and different treatment regimens, and was correlated with cardiac response. The higher the score, the more likely a cardiac response would occur.

**Conclusion:**

In conclusion, based on the large Chinese cohort of patients with AL and cardiac involvement, we identified nomogram models to predict cardiac response and OS. These models are more individualized and dynamic, and therefore, they have important clinical application value.

## Introduction

Light chain amyloidosis (AL) is an uncommon systemic disease (1). Abnormal light chain protein deposition and infiltration leads to dysfunction of target organs, including the heart, kidney, liver, and gastrointestinal tract. The incidence of heart involvement is reported to be almost 80% in AL patients (2). Cardiac amyloidosis typically causes restrictive cardiomyopathy which may involve life-threatening manifestations, such as progressive heart failure and fatal arrhythmias (3). Cardiac involvement is a crucial factor in terms of available treatment options and clinical prognosis (4). Despite recent improvements in the outcomes of AL with novel agents (5), the outcomes of advanced cardiac involvement remain dismal with a median survival time of only 6 months from the onset of heart failure (6). Cardiac involvement greatly affects the outcome; therefore, markers of cardiac injury and dysfunction are powerful prognostic factors in AL patients. At present, the most accepted system for estimating survival in patients with amyloidosis and cardiac involvement is the 2012 Mayo stage, which is based on cardiac troponin I (cTnI), N-terminal natriuretic peptide type B (NT-proBNP) and difference between involved and uninvolved free light chain (dFLC) levels (7). However, the 2012 Mayo stage does not take into account therapeutic factors, such as treatment regimen, cycle, and hematological response, that may influence the disease course and response to treatment. Moreover, the current prediction system cannot be used dynamically according to the patient’s individual condition. This study intends to establish an individualized evaluation system for patients with cardiac amyloidosis based on the initial state of the disease and the dynamics of the treatment process, and to evaluate the patient’s response to cardiac treatment and overall survival (OS).

Nomograms have been used as prediction tools to assess survival and prognosis of patients in multiple diseases, especially patients with tumors (8–10). A nomogram can be individualized to predict outcomes and has important clinical application value. To the best of our knowledge, only a few studies about nomograms for AL patients have been reported and no nomogram models have been established to predict cardiac involvement in AL patients. In the present study, novel nomograms for response and survival were developed based on a nationwide survey including 737 cases of AL with cardiac involvement. These models should be helpful for explaining the heterogeneity in the outcomes of AL patients with diverse treatments, thereby providing individual prediction of the probability of outcome events.

## Methods

### Patients

The study was approved by the Ethics Committee of Shengjing Hospital, China Medical University. The study was performed in accordance with the principles of the Declaration of Helsinki.

Through cooperation with 18 hospitals in the Chinese Registration Network for Light-chain Amyloidosis (CRENLA), a nationwide survey was conducted from 2009 to 2020, and 1269 patients with systemic AL were registered and followed. A total of 737 (58.1%) patients had cardiac involvement. The diagnosis of systemic AL was confirmed by the presence of Congo red-positive fibril deposition. Cardiac involvement was determined either by detection of amyloid deposits on endomyocardial biopsy or on histologic findings of a biopsy from other tissues in patients with abnormal cardiac findings including imaging and laboratory examinations.

We used the 2012 criteria for Mayo Clinic staging (11, 12). Hematologic and cardiac response after 3 cycles of treatment were assessed according to the Consensus Guidelines (13). All patients were followed up at our clinic or by telephone, and detailed follow-up data were recorded.

### Construction and Validation of the Nomogram

Of the 737 patients with cardiac involvement, the records of 553 patients were used to develop the nomogram models as training cohort. A validation cohort of 184 patients was used for further evaluation of the new prognostic models. Training cohort and validation cohort were randomly assigned in a 3:1 ratio. To develop the prognostic nomogram, the clinical features that have been shown to be associated with OS and cardiac response were incorporated: age, duration from onset to diagnosis, light chain, cTnI, dFLC, free light chain ratio (rFLC), Mayo classification 2012, New York Heart Association (NYHA) class, treatment regimen, courses of treatment, hematological response, and cardiac response. We used a univariate analysis to evaluate the prognostic factors for OS and cardiac response. Factors with a P value ≤0.05 were used in the multivariate proportional hazards model for OS and multivariate binary logistic regression model for cardiac response. With the nomograms, which were developed based on the multivariate model parameters, survival analysis was verified by C-index and cardiac response was verified by the area under the curve (AUC).

### Statistical Analysis

The baseline clinical features were evaluated using a c2 or nonparametric test, and univariate tests were performed using the Kaplan–Meier method with the log rank test for OS and univariate binary logistic regression for cardiac response. The multivariate proportional hazards model was used to assess the hazard ratios and the multivariate binary logistic regression model was used to assess the odds ratios. Two-sided P values <0.05 were considered statistically significant. All statistical analyses were conducted using SPSS version 23.0 (IBM Corp., Armonk, NY, USA). The nomogram and calibration curve were performed using the Hmisc, rms, and ggplot2 package in R software version 4.0.3 (R Project for Statistical Computing, Vienna, Austria). The final model selection for the nomograms was performed by a forward: LR selection process using a threshold of P.

## Results

### Baseline Characteristics

**Table 1** demonstrates the baseline characteristics of 737 patients with cardiac involvement and 532 patients without cardiac involvement. Of the 737 patients with cardiac systemic AL, 473 (64.2%) were male, and the median age was 59 years (range: 33–85 years). The median duration between onset and diagnosis was 9 months. Immunohistochemical staining identified 537 patients (72.86%) with lambda light-chain isotype and 174 patients (23.61%) with kappa light-chain isotype. Mayo (2012) Stage I, II, III, and IV disease was found in 10.99%, 18.32%, 28.22%, and 21.57% of patients, respectively. The median NT-proBNP and dFLC levels were 3773 ng/L (range: 34–103277) and 203.51 mg/L (0.274–12402.78), respectively. The median cTnI was 0.085 ug/L (0.0–1.945). For chemotherapy, bortezomib-containing treatment was administered to 403 (54.68%) patients and melphalan-containing treatment was administered to 64 (8.68%) patients. Sixty-nine patients (9.36%) chose immunomodulatory drugs, including thalidomide- and lenalidomide-based treatments. Only about 20% of all patients completed more than six courses of treatment.

**Table 1:** Baseline characteristics.

Variable	Total heart involvement (n = 737)	Derivation cohort (n = 553)	Validation cohort (n = 184)
Age, years, median (range)	59 (33–85)	59 (33–85)	60 (33–77)
Sex male: female	1.8:1 (473:261)	1.7:1 (349:203)	2.1:1 (124:58)
Duration between onset and diagnosis, months, median (range)	9 (0–146)	9 (0–146)	9 (0–144)
Involved light chains, n (%)			
Kappa	174 (23.61%)	131 (23.69%)	43 (23.37%)
Lambda	537 (72.86%)	404 (73.06%)	133 (72.28%)
Unrecorded	26 (3.53%)	18 (3.25%)	8 (4.35%)
NYHA class, n (%)			
1	146 (19.81%)	106 (19.17%)	40 (21.74%)
2	205 (27.82%)	156 (28.21%)	49 (26.63%)
3	201 (27.27%)	161 (29.11%)	40 (21.74%)
4	67 (9.0%)	45 (8.14%)	22 (11.96%)
Unrecorded	118 (16.01%)	85 (15.37%)	33 (17.93%)
Median NT-proBNP, ng/L, median (range)	3773 (34–103277)	3862.5 (34.0–103277.0)	3546.5 (91.0-33400.0)
Median cTnI, ug/L, median (range)	0.085 (0.0–1.945)	0.088 (0.0–1.945)	0.0785 (0.0–1.433)
Median dFLC, mg/L, median (range)	203.51 (0.274–12402.7)	194.59 (0.274–5939.6)	224.05 (0.5–12402.7)
Median rFLC, mg/L, median (range)	0.1221 (0.0–572.1154)	0.1205 (0.002–572.1154)	0.13575 (0.00–557.175)
Mayo Stage (2012), n (%)			
I	81 (10.99%)	58 (10.49%)	23 (12.50%)
II	135 (18.32%)	108 (19.53%)	27 (14.67%)
III	208 (28.22%)	153 (27.67%)	55 (29.89%)
IV	159 (21.57%)	122 (22.06%)	37 (20.11%)
Treatment			
Melphalan-containing	64 (8.68%)	48 (8.68%)	16 (8.70%)
Bortezomib-containing	403 (54.68%)	297 (53.71%)	106 (57.61%)
IMiD-containing	69 (9.36%)	51 (9.22%)	18 (9.78%)
Courses of treatment			
0	20 (2.71%)	14 (2.53%)	6 (3.26%)
1–3	228 (30.94%)	162 (29.29%)	66 (35.87%)
4–6	119 (16.15%)	89 (16.09%)	30 (16.30%)
7–9	139 (18.86%)	104 (18.81%)	35 (19.02%)
≥10	18 (2.44%)	14 (2.53%)	4 (2.17%)
Hematological response			
CR/VGPR/PR	288 (39.08%)	214 (38.70%)	74 (40.22%)
PD/NR/SD	92 (12.48%)	63 (11.39%)	29 (15.76%)
Cardiac response			
With response	177 (24.02%)	138 (24.95%)	39 (21.20%)
No response	560 (75.98%)	415 (75.05%)	145 (78.80%)
Survival			
Alive	437 (59.29%)	325 (58.77%)	112 (60.87%)
Dead	300 (40.71%)	228 (41.23%)	72 (39.13%)

NYHA, New York Heart Association; NT-proBNP; N-terminal natriuretic peptide type B; cTnI, cardiac troponin I; dFLC, uninvolved free light chain; rFLC, free light chain ratio; IMiD, immunomodulatory drug; CR, complete response; VGPR, very good partial response; PR, partial response; PD, partial disease; NR, no response; SD, stable disease.

The overall hematological response rate [complete response (CR) + very good partial response (VGPR) + partial response (PR)] after 3 cycles of treatment was 39.08% (288 cases), including 156 patients with CR (21.17%), 78 patients with VGPR (10.58%), and 54 patients with PR (7.33%). All 737 patients were evaluated for cardiac response after 3 cycles of treatment, and 177 (24.02%) achieved a cardiac response characterized by an NT-proBNP>30% and >300ng/L decrease in patients with baseline NT-proBNP ≥650ng/L or NYHA ≥2 class decrease in subjects with baseline NYHA class 3 or 4.

### Nomogram Model Developed to Predict Survival

Baseline demographic, laboratory diagnostic variables, and clinical treatment parameters for cardiac response and OS were further determined by univariate and multivariate Cox regression models. The result of univariate analysis showed that age, light chain type, NT-proBNP, cTnl, dFLC, rFLC, NYHA class, Mayo stage 2012, courses of treatment, therapeutic regimen, hematological response, and cardiac response were related to OS (P<0.05), as shown in **Table 2**. Subsequently, multivariate Cox regression analyses demonstrated that Mayo stage 2012, hematological response, cardiac response and courses of treatment were independently associated with OS.

**Table 2:** Univariate and multivariate analysis for factors associated with OS.

Characteristics	Univariate		Multivariate	
	HR (95% CI)	P-value	HR (95% CI)	P-value
**Age**	1.015 (1.000–1.030)	0.044		
**Duration between onset and diagnosis**	1.004 (0.998–1.011)	0.198		
**Involved light chains**		0.046		
AL-k	1			
AL-λ	0.744 (0.556–0.994)			
**NT-proBNP**	1.000 (1.000–1.000)	<0.001		
**cTnl**	2.205 (1.472–3.03)	<0.001		
**dFLC**	1.000 (1.000–1.000)	<0.001		
**rFLC**	1.003 (1.001–1.005)	0.002		
**NYHA**		<0.001		
I	1			
II	1.539 (0.969–2.444)			
III	2.803 (1.809–4.343)			
IV	4.686 (2.769–7.931)			
**Mayo Stage 2012**		<0.001		<0.001
I	1		1	
II	2.250 (1.079–4.694)		8.457 (1.100–65.017)	
III	3.466 (1.723–6.970)		7.505 (1.001–56.241)	
IV	5.415 (2.700–10.860)		26.401 (3.556–196.002)	
**Courses of treatment**		<0.001		0.007
Stage 1 (0–3)	1		1	
Stage 2 (4–6)	0.286 (0.186–0.439)		0.519 (0.248–1.082)	
Stage 3 (7–9)	0. 097 (0.053–0.177)		0.134 (0.042–0.430)	
Stage 4 (≥10)	0.108 (0.027–0.440)		0.461 (0.099–2.136)	
**Hematological response**		<0.001		0.001
CR/VGPR/PR	1		1	
PD/NR/SD	7.086 (4.637–10.829)		2.771 (1.493–5.144)	
**Cardiac response**		<0.001		0.001
With response	1		1	
No response	9.158 (5.227–16.047)		2.825 (1.198–6.660)	
**Treatment**		0.041		
Lenalidomide-containing	1			
Melphalan-containing	0.777 (0.374–1.612)			
Bortezomib-containing	0.497 (0.259–0.953)			
Thalidomide-containing	0.708 (0.326–1.537)			

CI, confidence interval; NT-proBNP; N-terminal natriuretic peptide type B; cTnI, cardiac troponin I; dFLC, uninvolved free light chain; rFLC, free light chain ratio; CR, complete response; VGPR, very good partial response; PR, partial response; PD, partial disease; NR, no response; SD, stable disease.

**Figure 1:**
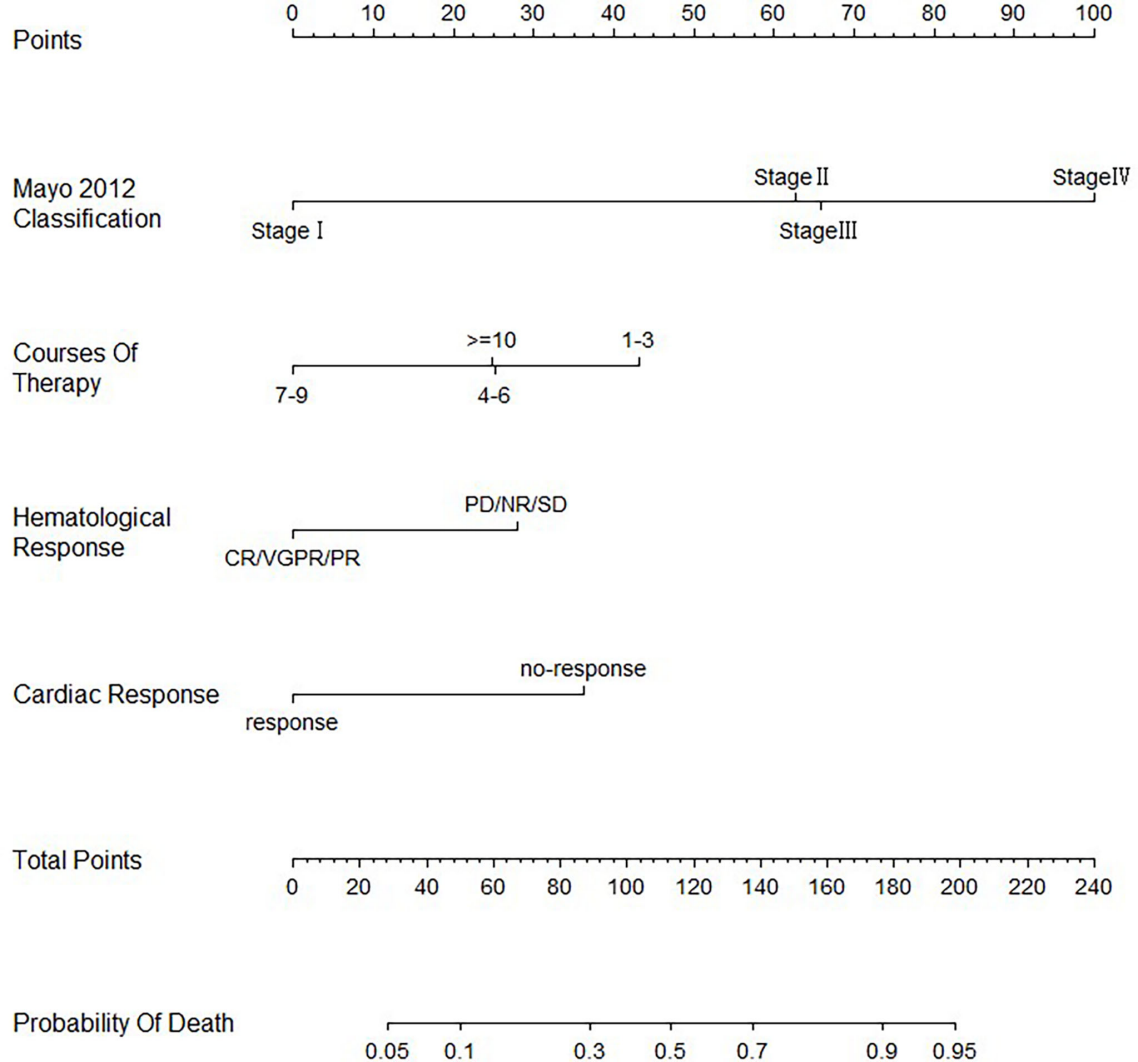
Nomogram for predicting overall survival in AL amyloidosis with cardiac involvement.

A nomogram including all four independent factors from the multivariate Cox proportional hazards analysis was constructed to calculate the possibility of achieving survival by adding all the points associated with the four variables (**Figure 1**). The higher the score, the more likely death would occur. The nomogram indicated that patients with early Mayo stage, administration of 7–9 courses of treatment, better hematological response, and cardiac response were correlated with better OS.

### Nomogram Model Developed to Predict Cardiac Response

In addition to survival time, we also hoped to predict the probability of a patient’s cardiac function remission. The result of the univariate analysis showed that light chain type, NT-proBNP, NYHA class, courses of treatment, therapeutic regimen, and hematological response were related to cardiac response (P<0.05), as shown in **Table 3**. Subsequently, multivariate Cox regression analyses demonstrated that hematological response, courses of treatment, and treatment regimen were independently associated with cardiac response.

**Table 3:** Univariate and multivariate analysis for factors associated with cardiac response.

Characteristics	Univariate		Multivariate	
	OR (95% CI)	P-value	OR (95% CI)	P-value
**Age**	0.993 (0.972–1.014)	0.498		
**Duration between onset and diagnosis**	0.993 (0.981–1.005)	0.252		
**Involved light chains**		0.049		
AL-k	1			
AL-λ	1.869 (1.134–3.078)			
**NT-proBNP**	1.000 (1.00–1.000)	<0.001		
**cTnl**	0.580 (0.233–1.445)	0.242		
**dFLC**	1.000 (1.00–1.000)	0.072		
**rFLC**	0.993 (0.983–1.002)	0.149		
**Mayo Stage 2012**		0.851		
I	1			
II	0.753 (0.376–1.508)			
III	0.882 (0.462–1.687)			
IV	0.929 (0.476–1.813)			
**NYHA**		0.011		
I	1			
Ii	1.965 (1.106–3.490)			
III	1.391 (0.775–2.498)			
IV	0.477 (0.168–1.352)			
**Treatment**		<0.001		0.098
IMiD-containing	1		1	
Melphalan-containing	1.314 (0.373–4.626)		0.994 (0.129–7.667)	
Bortezomib-containing	4.670 (1.566–17.572)		3.967 (0.774–20.345)	
**Courses of treatment**		<0.001		<0.001
Stage 1 (0–3)	1		1	
Stage 2 (4–6)	5.356 (2.829–10.141)		1.888 (0.711–5.015)	
Stage 3 (7–9)	23.565 (12.337–45.012)		8.860 (3.248–24.162)	
Stage 4 (≥10)	8.263 (2.614–26.116)		9.232 (1.433–59.455)	
**Hematological response**		<0.001		0.001
CR/VGPR/PR	1		1	
PD/NR/SD	56.14 (13.42–234.82)		0.067 (0.014–0.320)	

CI, confidence interval; NT-proBNP; N-terminal natriuretic peptide type B; cTnI, cardiac troponin I; dFLC, uninvolved free light chain; rFLC, free light chain ratio; IMiD, immunomodulatory drug; CR, complete response; VGPR, very good partial response; PR, partial response; PD, partial disease; NR, no response; SD, stable disease.

**Figure 2:**
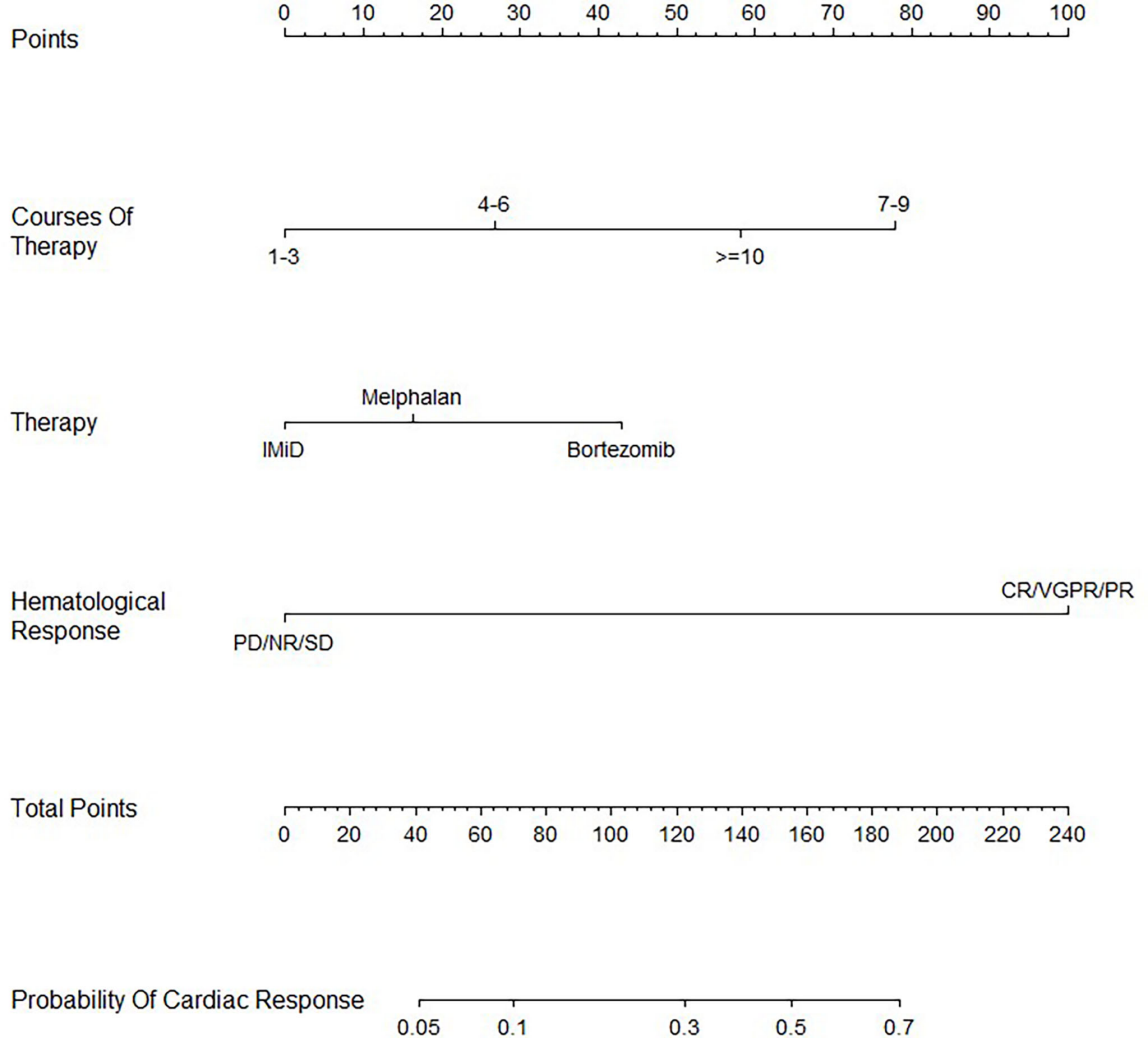
Nomogram for predicting cardiac response in AL amyloidosis with cardiac involvement.

A nomogram including all three independent factors from the multivariate Cox proportional hazards analysis was constructed to calculate the possibility of achieving cardiac response by adding all the points associated with the three variables (**Figure 2**). The higher the score, the more likely a cardiac response would occur. The nomogram indicated that 7–9 courses of treatment, better hematological response, and bortezomib-containing treatment were correlated with better cardiac response.

### Validation of the Nomograms

Validation of the nomograms was processed both internally and externally. Analysis of the internal validation cohort (training cohort) showed a C-index value of 0.871 for nomogram-based predictions of OS. Similarly, in the external validation cohort (validation cohort), the C-index value for predicting OS was 0.800. To validate the nomogram for prediction of cardiac response, the AUC values were calculated. Internal validation showed an AUC value of 0.816 for nomogram-based predictions of cardiac response and the external validation AUC was 0.826.

## Discussion

AL is a systemic disease which can damage multiple organs throughout the body. Cardiac involvement is the most common organ-related symptom of AL. The severity of cardiac involvement dictates the prognosis in AL. Currently the Mayo scoring system 2004 or 2012 is widely used for assessing amyloidosis and it mainly predicts survival based on indicators of early myocardial damage, without considering the impact of treatment factors on survival. Moreover, there is no model that can predict the effectiveness of cardiac therapy. Therefore, the purpose of this study was to establish a model for predicting survival and a model for predicting cardiac responsiveness using the largest amyloidosis heart involvement database in China, so as to better guide clinical treatment.

Nomograms are pictorial representations of a complex mathematical formula which are widely used as prognostic devices in the field of medicine. By integrating biological and clinical variables, medical nomograms generate a simple graphical representation of a statistical predictive model and create a numerical probability of a clinical event, such as death (14, 15). Therefore, nomograms can contribute to modern medical decision-making by providing estimated prognosis reliably and conveniently. Moreover, through the nomogram model, each patient will have a different, individualized score calculated to predict the probability of clinical outcomes. This result is unique. In the present study, we developed two nomogram models, one for predicting the OS of AL with cardiac involvement, and the other for predicting the cardiac response.

Among the factors used in the nomogram model to predict OS, four indicators were considered. The first was the Mayo staging system that has been widely used for prediction of OS. Our model then adds three treatment-related factors, which are the courses of treatment, whether hematological remission was achieved after treatment, and cardiac remission. Notably, our data further showed that patients achieving cardiac response have better prognosis compared to patients with no cardiac response. It indicated that not only hematologic response, but also heart function remission, finally translate into survival benefits. According to our model, 7–9 courses of treatment are the most suitable. A greater number of courses of treatment will not bring about additional survival benefits. It may be that more courses of treatment may lead to drug-related toxicity. On the contrary, a lower number of courses of treatment cannot achieve the maximum effect of the treatment. The key point is to explore the optimal dose and treatment interval in order to balance the efficacy and treatment-related adverse effects. If the patient quickly obtains hematological remission and cardiac remission after treatment, this indicates a better prognosis. This combines better efficacy indicators with prognosis. At the same time, the nanogram model quantifies and accumulates multiple factors simultaneously to obtain individualized predictive values, which is more clinically meaningful than the current commonly used single staging system.

Currently, very few published series have described the determinants of cardiac response. Our nomogram prediction model is mainly based on the proportion of patients who already have cardiac involvement. It can be seen that the choice of treatment is of great significance to the function of the heart. First, from the point of view of drug selection, the treatment program based on bortezomib is the most effective for obtaining cardiac remission, followed by the program based on melphalan. Bortezomib-based regimens have been the standard therapy for AL and are recommended by both the National Comprehensive Cancer Network and UK guidelines (16) despite the fact that no randomized clinical trial has shown the efficacy and safety of bortezomib-including regimens to date. One European study previously reported the efficacy of bortezomib-cyclophosphamide-dexamethasone (CyBorD) for the initial treatment in 230 patients with AL. The overall hematologic response rate was 60% (23% complete) and cardiac response was 17% (17). In the treatment of AL, the effect of immunomodulatory drugs on cardiac involvement and recovery seems to be limited (18). Both lenalidomide and thalidomide are immunomodulatory derivatives. In a trial of lenalidomide, melphalan, and dexamethasone with 22 of 25 patients having stage II or III cardiac amyloidosis, the overall hematologic response rate was 58% and organ responses were seen in only 8% of patients. Cardiac arrhythmias were seen in 33% (18). Indeed, lenalidomide is frequently observed to be associated with the accumulation of NT-proBNP, and the mechanisms remain unknown (19, 20). Use of thalidomide has been shown to improve response to therapy, but also increases the risk of toxicities (21). The choice of treatment course also shows that 7–9 courses of treatment is most suitable, and more courses of treatment will not bring additional benefits of cardiac response, which also verifies the current view of AL with a fixed course of treatment. Deep relief of hematology brings about benefits in terms of cardiac relief. We integrated a variety of treatment-related factors to predict the success rate of cardiac treatment. This result has important clinical application value.

From our model, different treatment options do not bring about statistically significant OS outcomes, which also suggests that for AL patients with cardiac involvement, more effective treatment options still need to be explored. Daratumumab is an anti-CD38 monoclonal antibody used for multiple myeloma. Daratumumab has been reported to have good effects on AL (22–24). Among patients with newly diagnosed AL, the addition of daratumumab to bortezomib, cyclophosphamide, and dexamethasone was associated with higher rates of hematologic response than a control group (53.3% vs. 18.1%). At 6 months, more cardiac responses occurred in the daratumumab group than in the control group (41.5% vs. 22.2%) (25). In addition, ANDROMEDA study showed Daratumumab subcutaneous(DARA SC)-CyBorD was well tolerated in patients of AL amyloidosis, and importantly, with high rates of deep and durable hematologic and organ responses (24). However, Daratumumab is new antibody drug and the sample size treated with Daratumumab in our database is insufficient to be included in the nomogram model. With the increase in data, our nanogram will continue to incorporate new treatment options for adjustments in the future to better predict clinical outcomes.

In conclusion, based on the largest Chinese cohort of patients with cardiac AL, we identified a nomogram model to predict cardiac response and OS in AL patients with cardiac involvement. This model is more individualized and dynamic, it can accurately predict the clinical outcomes, and it has important clinical application value.

## Data Availability Statement

The raw data supporting the conclusions of this article will be made available by the authors, without undue reservation.

## Ethics Statement

Written informed consent was obtained from the individual(s) for the publication of any potentially identifiable images or data included in this article.

## Author Contributions

YL, HW, and JL designed and organized the study. YL and HW wrote the first draft of the manuscript. MZ and YC analyzed the data statistically. WYan, AL, and WYang revised the manuscript critically. All authors contributed to the article and approved the final version of the manuscript.

## Funding

This study received technical support from Neusoft Co., Ltd. Neusoft Co., Ltd. was not involved in the study design, collection, analysis, interpretation of data, the writing of this article or the decision to submit it for publication. There was no external funding for this study.

## Conflict of Interest

The authors declare that the research was conducted in the absence of any commercial or financial relationships that could be construed as a potential conflict of interest.

This study received technical support from Neusoft Co., Ltd. It had the following involvement with the study: the data was processed and analyzed on their automatic medical analysis platform.

## Publisher’s Note

All claims expressed in this article are solely those of the authors and do not necessarily represent those of their affiliated organizations, or those of the publisher, the editors and the reviewers. Any product that may be evaluated in this article, or claim that may be made by its manufacturer, is not guaranteed or endorsed by the publisher.
